# The deubiquitinating enzyme OTUD1 antagonizes BH3-mimetic inhibitor induced cell death through regulating the stability of the MCL1 protein

**DOI:** 10.1186/s12935-019-0936-5

**Published:** 2019-08-27

**Authors:** Lanqin Wu, Yingying Lin, Jinan Feng, Yuanlin Qi, Xinrui Wang, Qiaofa Lin, Wanyan Shi, Enrun Zheng, Wei Wang, Zhenzhu Hou, Hanbin Lin, Cheng Yu, Yan He, Yan Xu, Hong Yang, Ling Lin, Lisheng Li

**Affiliations:** 1 The School of Basic Medical Sciences, Fujian Medical University, Minhou, Fuzhou China; 2 State Key Laboratory for Medical Genomics, Shanghai Institute of Hematology, Rui Jin Hospital Affiliated to School of Medicine, Shanghai Jiao Tong University, Shanghai, China; 3 Key Laboratory of Ministry of Education for Gastrointestinal Cancer, Fujian Medical University, 1 Xueyuan Road, Minhou, Fuzhou China

**Keywords:** Deubiquitinating enzyme, OTUD1, MCL1, BH3-mimetic inhibitor

## Abstract

**Background:**

Myeloid cell leukaemia 1 (MCL1) is a pro-survival Bcl-2 family protein that plays important roles in cell survival, proliferation, differentiation and tumourigenesis. MCL1 is a fast-turnover protein that is degraded via an ubiquitination/proteasome-dependent mechanism. Although several E3 ligases have been discovered to promote the ubiquitination of MCL1, the deubiquitinating enzyme (DUB) that regulates its stability requires further investigation.

**Methods:**

The immunoprecipitation was used to determine the interaction between OTUD1 and MCL1. The ubiquitination assays was performed to determine the regulation of MCL1 by OTUD1. The cell viability was used to determine the regulation of BH3-mimetic inhibitor induced cell death by OTUD1. The survival analysis was used to determine the relationship between OTUD1 expression levels and the survival rate of cancer patients.

**Results:**

By screening a DUB expression library, we determined that the deubiquitinating enzyme OTUD1 regulates MCL1 protein stability in an enzymatic-activity dependent manner. OTUD1 interacts with MCL1 and promotes its deubiquitination. Knockdown of OTUD1 increases the sensitivity of tumour cells to the BH3-mimetic inhibitor ABT-263, while overexpression of OTUD1 increases tumour cell tolerance of ABT-263. Furthermore, bioinformatics analysis data reveal that OTUD1 is a negative prognostic factor for liver cancer, ovarian cancer and specific subtypes of breast and cervical cancer.

**Conclusions:**

The deubiquitinating enzyme OTUD1 antagonizes BH3-mimetic inhibitor induced cell death through regulating the stability of the MCL1 protein. Thus, OTUD1 could be considered as a therapeutic target for curing these cancers.

## Background

The B cell lymphoma 2 (BCL2) family plays a central role in the intrinsic apoptotic response. The BCL2 family members can be separated into anti-apoptotic proteins and pro-apoptotic proteins according to their different roles in apoptosis. The anti-apoptotic proteins, including BCL2, BCLXL, BCLW, MCL1 and A1, protect cells from chemotherapy drug-induced apoptosis and are dysregulated in many cancers. Among these proteins, MCL1 is a highly unstable protein under normal conditions. The stability of MCL1 protein is mainly regulated by an ubiquitination/proteasome-dependent mechanism. MCL1 is ubiquitinated by several E3 ubiquitin (Ub) ligases, including MULE and FBW7 [1–3]. By targeting MCL1 for ubiquitination and degradation, or by knocking it down, cancer cells are highly sensitized to chemotherapy drugs [2, 3]. Therefore, developing a highly potent and selective small-molecule inhibitor of the pro-survival protein MCL1 will provide a suitable therapeutic scheme for cancer patients [4]. Although E3 ligases of MCL1 protein have been reported, the deubiquitinating enzymes (DUBs) that participate in the regulation of MCL1 stability still need further investigation.

DUBs play key roles in regulating ubiquitinated proteins and are important to maintain the balance between ubiquitination and deubiquitination. The human genome encodes approximately 95 putative DUBs [5], and DUBs have been implicated in diverse signalling pathways through regulating the stability of many proteins. Accumulating evidences indicates that DUBs play important roles in cancer development and progression including cell-cycle regulation, DNA damage repair, chromatin remodelling, and tumor relative signalling pathway [6]. Therefore, DUBs are considered as potential anticancer targets and several inhibitors of DUBs have been developed [7]. However, the physiological or pathological roles of DUBs still need to further investigate.

In the present study, we screened a deubiquitinating enzyme expression library to identify the DUBs that regulate MCL1 stability. We found that OTU domain containing protein 1 (OTUD1) regulated the stability of MCL1 in an enzymatic-activity dependent manner. We also found that OTUD1 interacted with MCL1 and removed the ubiquitin chain from it. Knockdown of OTUD1 reduced the levels of MCL1 and sensitized cells to the BH3-mimetic compound ABT-263. Based on our findings, OTUD1 deubiquitinates MCL1 and regulates its downstream cellular functions. Taken together, our results indicate a novel and critical role of OTUD1 in regulating MCL1 protein stability.

## Materials and methods

### Cell culture

The HEK293T, MCF7, Huh7, and HeLa cell lines were purchased from the Cell Bank of the Chinese Academy of Sciences (Shanghai, China). MCF-10A cell line was purchased from Guangzhou Cellcook Biotech Co., Ltd (Guangzhou, China). All of the cells were maintained in Dulbecco’s Modified Eagle’s Medium (DMEM) supplemented with 10% foetal bovine serum, 4 mM l-glutamine, 100 IU penicillin and 100 mg/ml streptomycin at 37 °C in a humidified incubator containing 5% CO_2_.

### Reagents

Mouse anti-HA (F-7), mouse anti-GAPDH (6C5) and mouse anti-β-actin (C4) antibodies were obtained from Santa Cruz Biotechnology, Inc. (Santa Cruz, CA, USA). The mouse anti-flag antibody and protease inhibitor cocktail were obtained from Sigma (Saint Louis, MO, USA). The MCL1 and ubiquitin antibodies were obtained from Proteintech Company. The CellTiter-Glo Luminescent Cell Viability Assay Kit and Caspase-Glo^®^ 3/7 Assay Kit were obtained from Promega Company. MG-132 was obtained from Merck Millipore Company. ABT-263 was obtained from Selleck Company. Sorafenib was obtained from MCE Company. The TRIZOL reagent was obtained from Invitrogen. The Top Green qPCR SuperMix was obtained from Transgen Biotech.

### Ubiquitination assays

To assess MCL1 ubiquitination in vivo, 293T cells were co-transfected with MCL1-HA and FLAG-UB, with or without OTUD1 or its enzymatic activity mutant C320S plasmids. After lysis in RIPA buffer, MCL1 was immunoprecipitated with anti-HA beads. The immunoprecipitated MCL1-HA was subjected to SDS/PAGE and the immunoblots were probed with the indicated antibodies.

### RNA extraction and real-time PCR analysis

The RNA extractions and real-time PCR analyses were performed as previously described [8, 9]. Total RNA was isolated from the cells using TRIZOL reagent, according to the manufacturer’s instructions. Each RNA sample was used to prepare cDNAs by reverse transcription using oligo(dT) primers. The RNA expression levels were normalized to an internal control, GAPDH. Real-time PCR was performed in a Stratagene Mx3000P System (Agilent Technologies).

### Immunoprecipitation

Protein interactions were assessed using immunoprecipitation. Briefly, cells were washed once with cold PBS and lysed in cold lysis buffer containing protease inhibitors. The supernatants of the cell lysates were isolated by centrifugation at 12,500×*g* for 30 min at 4 °C. Immunoprecipitation was performed using anti-flag M2 beads or anti-HA beads as previously described. For the ubiquitin immunoprecipitation assay, the cells were lysed in RIPA buffer containing protease inhibitors. The anti-HA beads were washed with RIPA buffer. The other steps were similar to typical immunoprecipitation assays for protein interactions.

### Cell viability determination

Determination of cell viability was performed following the kit manual. Briefly, approximately 2.5 × 10^4^ cells were seeded into a 96-well plate and incubated overnight. The cells were treated with or without relevant compounds for the proper times. Then, the 96-well plate and its contents were maintained at room temperature for 30 min. An equal volume of CellTiter-Glo^®^ Reagent was added into the cell culture medium and incubated for 2 min on a shaker. The 96-well plate was further incubated for 10 min at room temperature and the luminescence was recorded.

### Survival analysis

Gene expression levels and clinical survival data of 365 liver hepatocellular carcinoma (LIHC) patients were obtained from the TCGA dataset. For the survival analysis, all of the LIHC patients were divided into the low-expression or high-expression groups based on the cut-off values of gene expression. Overall survival (OS) distributions of the LIHC patients were estimated using Kaplan–Meier analysis and the p-values were calculated using the log rank test.

## Results

### OTUD1 regulates MCL1 protein stability

**Fig. 1:**
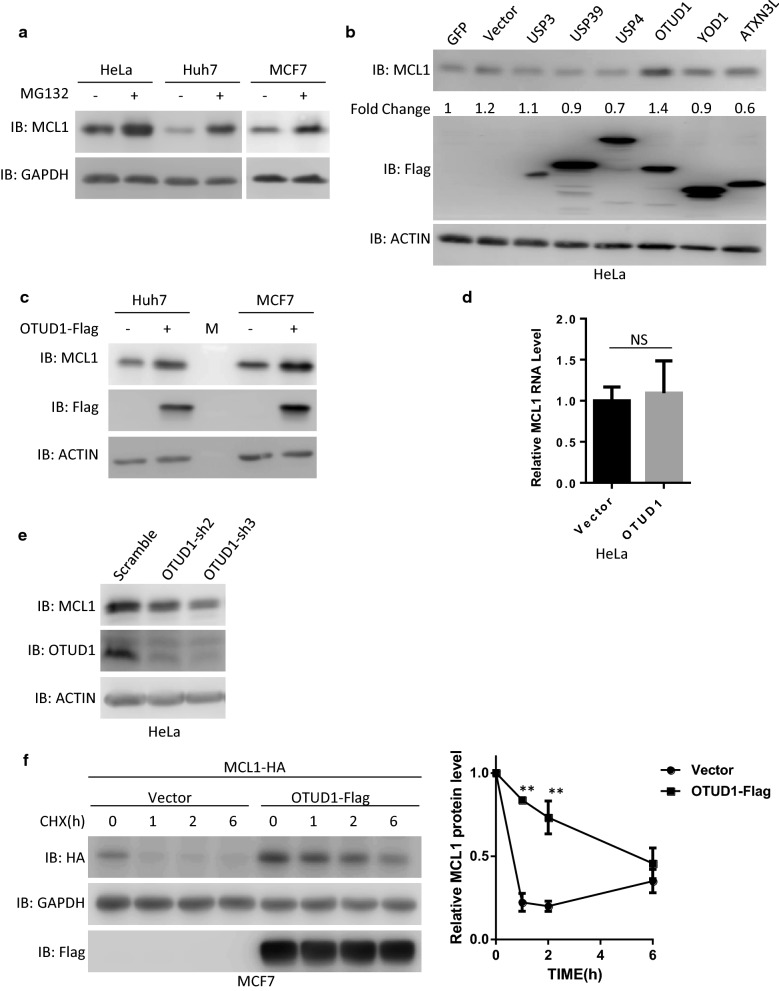
OTUD1 regulates MCL1 protein stability. **a** Different cancer cell lines were treated with the proteasome inhibitor MG132 (10 µM) for 6 h. The cells were then harvested and subjected to SDS-PAGE and analysed by immunoblotting (IB) with the indicated antibodies. **b** Individual DUB expression plasmids were transfected into HeLa cells. Then, 36 h later, the cells were harvested and subjected to SDS-PAGE and analysed by immunoblotting with the indicated antibodies. **c** The empty vector or a plasmid expressing OTUD1 was transfected into Huh7 cells or MCF7 cells. Then, 36 h later, whole cellular extracts were subjected to Western blotting. **d** MCL1 RNA levels were determined in HeLa cells transfected with the empty vector or a plasmid expressing OTUD1. NS: no significant difference. **e** HeLa cells were infected with lentivirus expressing scramble shRNA or OTUD1-shRNA. Then, the cells were harvested and subjected to SDS-PAGE and analysed by immunoblotting with the indicated antibodies. **f** The MCL1-HA-expressing plasmid was co-transfected with the empty vector or a plasmid expressing OTUD1 into MCF7 cells. Then, 24 h later, the cells were treated with CHX for the indicated times. Next, the cells were harvested and subjected to SDS-PAGE and analysed by immunoblotting with the indicated antibodies. The intensities of the MCL1-HA expression levels were quantified by densitometry and plotted. The experiment was repeated three times, and a representative experiment is presented. **p < 0.01

Previous studies indicated MCL1 is a highly unstable protein in many cells [1, 10]. Consistent with these data, we found that MCL1 protein levels were significantly upregulated in cells pretreated with the proteasome inhibitor MG132 (Fig. 1a and Additional file 1: Fig. S1A), which indicated that MCL1 was degraded in an ubiquitination/proteasome-dependent manner in these cells. Then, we transfected the individual DUBs into HeLa cells to identify whether DUBs can upregulate MCL1 protein levels. We found that overexpression of OTUD1 significantly increased MCL1 protein levels (Fig. 1b). Surprisingly, USP13, a previous reported deubiquitinating enzyme of MCL1 protein, did not increased MCL1 protein levels in Hela cells, which indicated different deubiquitinating enzymes regulated MCL1 protein level in a cell type dependent manner (Additional file 1: Fig. S1B). Moreover, OTUD1 also increases MCL1 protein levels in the MCF7 breast cancer cell line and the Huh7 liver cancer cell line (Fig. 1c and Additional file 1: Fig. S1C). In contrast, overexpression of OTUD1 did not influence the RNA levels of MCL1 (Fig. 1d). We next tested whether knockdown of OTUD1 influenced MCL1 protein levels. As shown in Fig. 1e and Additional file 1: Fig. S1D, knockdown OTUD1 significantly reduced protein levels of OTUD1 and MCL1 without influencing the cell viability. To further confirm that OTUD1 regulated MCL1 protein levels, we co-transfected OTUD1 and MCL1 into HeLa cells and then treated them with cycloheximide (CHX). As shown in Fig. 1f, we found that MCL1 protein stability was increased in OTUD1-overexpressing cells. We also detected OTUD1 and MCL1 expression levels in different cell lines and found that high protein level of MCL1 is related to high protein level of OTUD1 (Additional file 1: Fig. S1E). Taken together, OTUD1 regulates MCL1 protein stability.

### OTUD1 regulates MCL1 protein stability in an enzymatic activity-dependent manner

**Fig. 2:**
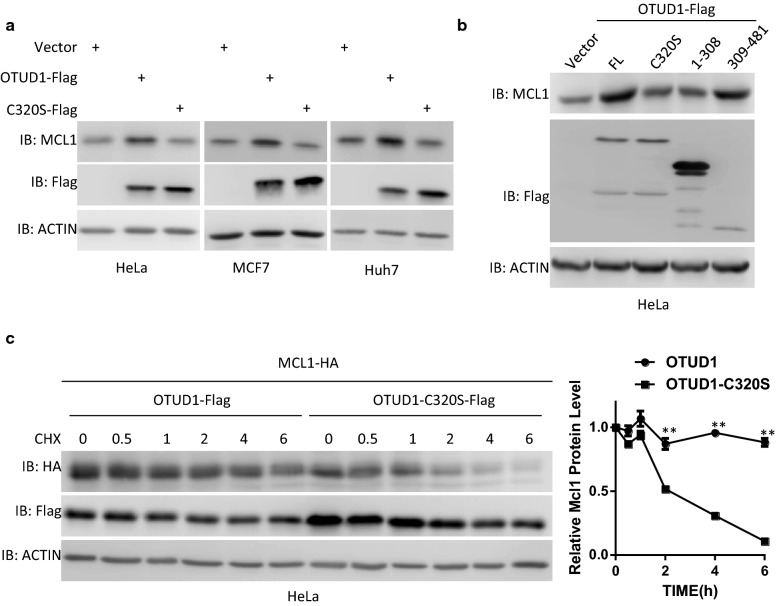
OTUD1 regulates MCL1 protein stability in an enzymatic activity-dependent manner. **a** The indicated cells were infected with lentivirus expressing empty vector, OTUD1 or its enzymatic activity mutant C320S. Then, 36 h later, whole cellular extracts were subjected to Western blotting. **b** HeLa cells were infected with lentivirus expressing empty vector, OTUD1 or its functional domains. Then, 36 h later, whole cellular extracts were subjected to Western blotting. **c** The MCL1-HA-expressing plasmid was co-transfected with a plasmid expressing OTUD1 or its enzymatic activity mutant C320S into HeLa cells. Then, 24 h later, the cells were treated with CHX for the indicated times. The cells were then harvested and subjected to SDS-PAGE and analysed by immunoblotting with the indicated antibodies. The intensities of the MCL1-HA expression levels were quantified by densitometry and plotted. The experiment was repeated three times, and a representative experiment is presented. **p < 0.01

The enzymatic activity of DUBs is important for their function. Therefore, we tested whether the enzymatic activity of OTUD1 is required for its regulation of MCL1 protein stability. Mutation of Cys320 into Ser (C320S) abolished the enzymatic activity of OTUD1 [11]. As shown in Additional file 1: Fig. S2A, when co-transfected with MCL1, OTUD1, but not the enzymatic activity mutant OTUD1-C320S, increases exogenous MCL1 protein levels in different cells. Similarly, OTUD1, but not its enzymatic activity mutant OTUD1-C320S, increases the endogenous MCL1 protein levels (Fig. 2a and Additional file 1: Fig. S2B). These data also indicated the regulation of MCL1 protein levels by OTUD1 is independent of exogenous or endogenous promoter activity. Consistently, we found that the enzyme domain of OTUD1 (amino acids 309-481) is more sufficient to increase MCL1 protein levels than OTUD1 1-308 domain, even though the expression level of the enzyme domain of OTUD1 is significantly lower than OTUD1 1-308 domain (Fig. 2b and Additional file 1: Fig. S2C). Moreover, compared with OTUD1-C320S, only wild-type OTUD1 prolonged the half-life of MCL1 (Fig. 2c). Taken together, the enzymatic activity of OTUD1 is required for its regulation of MCL1 stability.

### OTUD1 interacts with MCL1 and promotes MCL1 deubiquitination

**Fig. 3:**
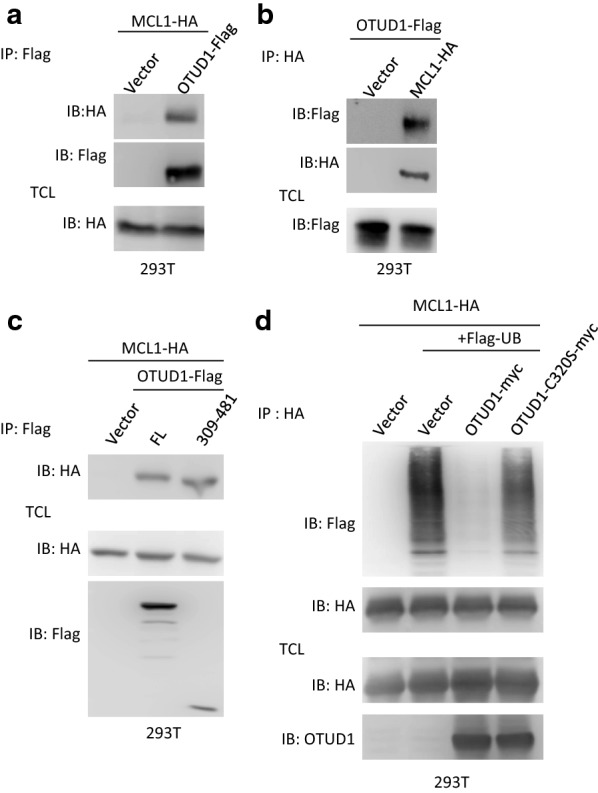
OTUD1 interacts with MCL1 and promotes MCL1 deubiquitination. **a** The indicated plasmids were transfected into 293T cells. Next, the cell lysates were immunoprecipitated with an anti-Flag antibody. The lysates (TCL) and the immunoprecipitates were immunoblotted with the indicated antibodies. **b** The indicated plasmids were transfected into 293T cells. The cell lysates were then immunoprecipitated with an anti-HA antibody. The lysates (TCL) and the immunoprecipitates were immunoblotted with indicated antibodies. **c** The empty vector, OTUD1 or its enzymatic domain (amino acids 309-481) were co-transfected with MCL1-HA into 293T cells. The cell lysates were immunoprecipitated with an anti-Flag antibody. The lysates (TCL) and the immunoprecipitates were immunoblotted with the indicated antibodies. **d** The empty vector, OTUD1 or its enzymatic activity mutant C320S-expressing plasmid was co-transfected with MCL1-HA and Flag-ubiquitin expression plasmids into 293T cells. The cells were treated with MG132 (10 µM) for 6 h, and then the cells were lysed in RIPA buffer and the cell lysates were immunoprecipitated with anti-HA antibody. The lysates and the immunoprecipitates were subjected to SDS-PAGE and analysed by immunoblotting with the indicated antibodies

Next, we tested whether OTUD1 interacts with MCL1. As shown in Fig. 3a, Additional file 1: Fig. S3A, B, and S3B, OTUD1 interacts with MCL1 in both directions when co-transfected into cells. Furthermore, we found that the enzyme domain of OTUD1 (amino acids 309–481) is sufficient to interacts with MCL1 (Fig. 3c and Additional file 1: Fig. S3C), which is consistent with our previous data (Fig. 2b). Since OTUD1 interacts with MCL1 and its enzymatic activity is required for MCL1 stability, then we tested whether OTUD1 influences the ubiquitination of MCL1. As shown in Fig. 3d and Additional file 1: Fig. S3D, OTUD1, but not its enzymatic activity mutant C320S, deubiquitinated MCL1. These results indicate that OTUD1 interacts with MCL1 and regulates its stability through deubiquitination.

### OTUD1 regulates sensitivity to BH3 mimetic compound in cancer cells

**Fig. 4:**
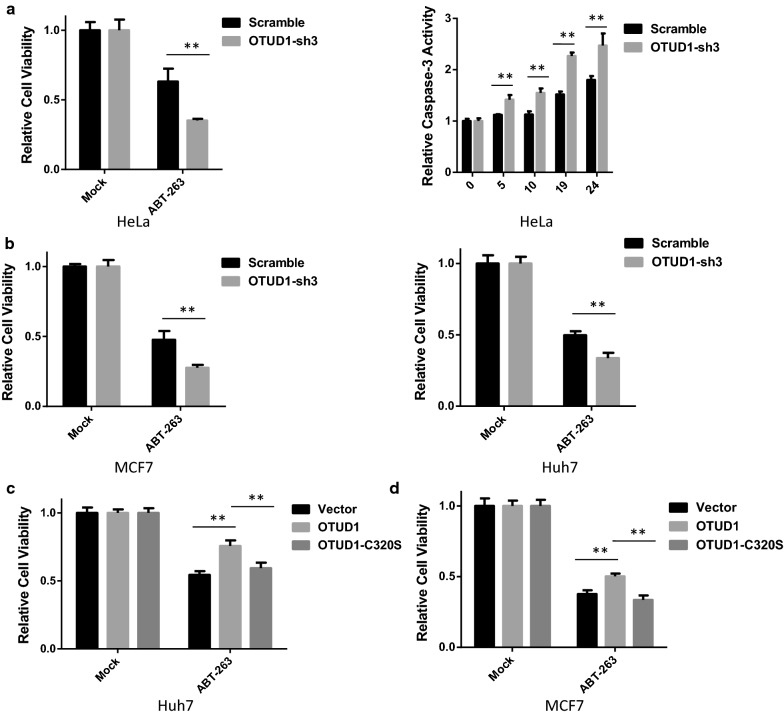
OTUD1 regulates sensitivity to a BH3-mimetic compound in cancer cells. **a** HeLa cells were infected with a scramble or an OTUD1-shRNA lentivirus to generate stable cell lines. The HeLa cells were then seeded into 96-well plates and treated with the BH3-mimetic compound ABT-263 (10 µM). The cell viabilities were determined using the CellTiter-Glo Luminescent Cell Viability Assay method. The caspase3/7 activities were measured using the Caspase-Glo^®^ 3/7 Assay. **b** MCF7 and Huh7 cells were infected with a scramble or an OTUD1-shRNA lentivirus to generate stable cell lines. Then, both cell lines were seeded into 96-well plates and treated with the BH3-mimetic compound ABT-263, and the cell viabilities were determined. **c** Huh7 cells were infected with empty vector, OTUD1 or OTUD1-C320S lentiviruses to generate stable cell lines. Then, the Huh7 cell line was seeded into 96-well plates and treated with the BH3-mimetic compound ABT-263 (15 µM) and the cell viabilities were determined. **d** The same procedure was followed as described in **c**, except the cells tested are MCF7 cells. **p < 0.01

The BH3 mimetics ABT-737 and ABT-263 selectively target BCL-XL, BCL-2, and BCL-W, but not MCL1 or A1 [12]. The sensitivity of cancer cells to BH3 mimetics is highly dependent on the protein level of MCL1 [13, 14]. As OTUD1 regulates MCL1 protein levels, we tested whether OTUD1 influences the toxicity of a BH3 mimetic in cancer cells. We found that stimulation of BH3 mimetics ABT-263 induced cell death in the cell lines we tested (Additional file 1: Fig. S4A). As shown in Fig. 4a, knockdown of OTUD1 significantly potentiated ABT-263-induced cell death in HeLa cells. Similar results were observed in Huh7 and MCF7 cells (Fig. 4b and Additional file 1: Fig. S4B). Moreover, overexpression of OTUD1, but not its enzymatic activity mutant C320S, reduced the toxicity of ABT-263 in Huh7 and MCF7 cells (Fig. 4c, d). Several reports indicated OTUD1 also regulates p53 and SMAD7 protein stability. However, we found that OTUD1 did not increase p53 and SMAD7 in the cell line we tested, which indicated OTUD1 regulates sensitivity to BH3 mimetic compound in cancer cells is independent of p53 and SMAD7 (Additional file 1: Fig. S4C). Taken together, these results show that OTUD1 regulates sensitivity to a BH3-mimetic compound in different cancer cells.

### OTUD1 attenuates the synergistic effect of Sorafenib and BH3 mimetic compound in cancer cells

**Fig. 5:**
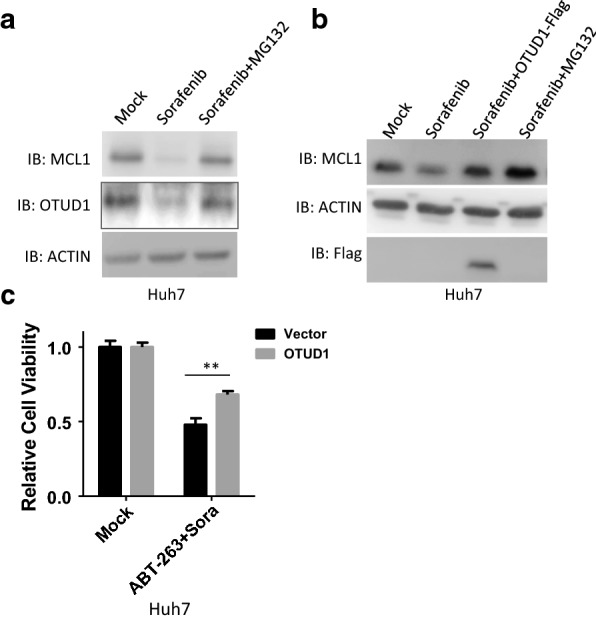
OTUD1 attenuates the synergistic effect of Sorafenib and BH3 mimetic compound in cancer cells. **a** Huh7 cells were stimulated with or without Sorafenib (10 µM) for 6 h. The cells were then harvested and subjected to SDS-PAGE and analysed by immunoblotting with the indicated antibodies. **b** Huh7 stable cell lines expressing empty vector or OTUD1 were treated with or without Sorafenib (10 µM) for 3 h. The cells were then harvested and subjected to SDS-PAGE and analysed by immunoblotting with the indicated antibodies. **c** Huh7 stable cell lines expressing empty vector or OTUD1 were treated with Sorafenib (2 µM) plus ABT-263 (5 µM) and the cell viabilities were determined. **p < 0.01

Sorafenib is the first-line FDA-approved drug for advanced hepatocellular carcinoma (HCC) [15]. Sorafenib increases BH3-mimetic compound-induced cell death through downregulating MCL1 [16–19]. As shown in Fig. 5a and Additional file 1: Fig. S5A, Sorafenib induced degradation of MCL1 protein, which can be reversed by proteasome inhibitor MG132. Interestingly, Sorafenib also induced OTUD1 degradation in a proteasome dependent manner (Fig. 5a and Additional file 1: Fig. S5A). Then we tested whether overexpression of OTUD1 can reverse the downregulation of MCL1 induced by Sorafenib. As shown in Fig. 5b and Additional file 1: Fig. S5B, OTUD1 attenuated the downregulation of MCL1 induced by Sorafenib. We also found that treatment of Sorafenib induced cell death in the cell lines we tested (Additional file 1: Fig. S5C). Moreover, we found that Sorafenib increases BH3-mimetic compound-induced cell death (Additional file 1: Fig. S5D), which is consistent with previous reports [20, 21]. Consistent with our findings, OTUD1 reduced the toxicity induced by the combination of Sorafenib and ABT-263 (Fig. 5c). Taken together, these results show that OTUD1 attenuates the synergistic effect of Sorafenib and BH3 mimetic compound in cancer cells.

### OTUD1 is a negative prognostic factor for multiple cancers

**Fig. 6:**
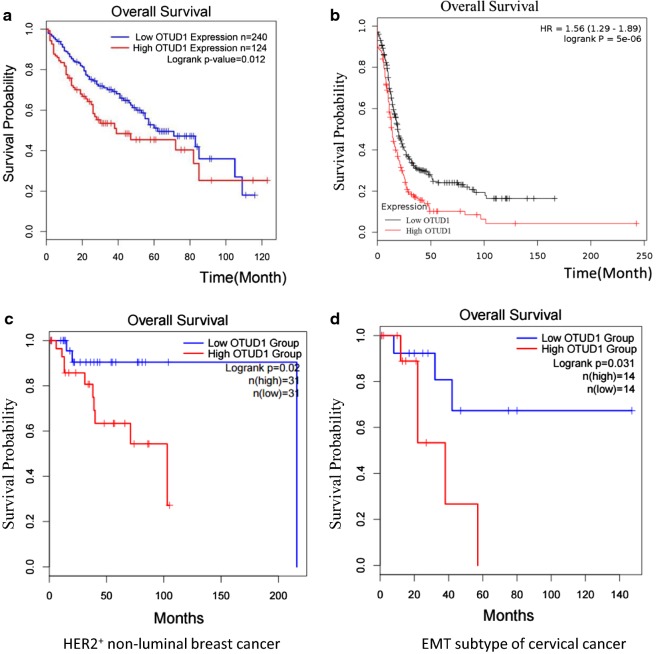
OTUD1 is a negative prognostic factor for liver cancer and ovarian cancer. **a** The Kaplan–Meier analysis of OTUD1 expression in liver hepatocellular carcinoma using the TCGA database was performed. High expression of OTUD1 was correlated with poorer prognosis in liver cancer (LIHC). **b** Kaplan–Meier curves of recurrence-free survival of patients with ovarian cancer, stratified by OTUD1 expression levels. The data were obtained from http://kmplot.com/analysis/. **c** The Kaplan–Meier analysis of OTUD1 expression in HER2^+^ non-luminal breast cancer using the TCGA database was performed. High expression of OTUD1 was correlated with poorer prognosis in HER2^+^ non-luminal breast cancer. **d** The Kaplan–Meier analysis of OTUD1 expression in EMT subtype of cervical cancer using the TCGA database was performed. High expression of OTUD1 was correlated with poorer prognosis in EMT subtype of cervical cancer

MCL1 was expected to be a negative prognostic factor in many cancers [22–25]. Next, we tested the relationship between OTUD1 expression levels and the survival rate of cancer patients. As shown in Fig. 6a, analysis of the TCGA database revealed that patients with high OTUD1 expression showed poorer overall survival than patients with low OTUD1 expression. We also performed Kaplan–Meier (KM) plotter analysis of microarray datasets of human ovarian tumours [26]. As shown in Fig. 6b, OTUD1 is also a negative prognostic factor for ovarian cancer patients. Furthermore, higher OTUD1 expression was significantly associated with low recurrence-free survival of HER2^+^ non-luminal subtype of breast cancer patients and EMT subtype of cervical cancer patients (Fig. 6c, d). Thus, OTUD1 is a negative prognostic factor for liver cancer, ovarian cancer, specific subtypes of breast and cervical cancer. We proposed that OTUD1 could be considered as a therapeutic target for curing these cancers.

## Discussion

**Fig. 7:**
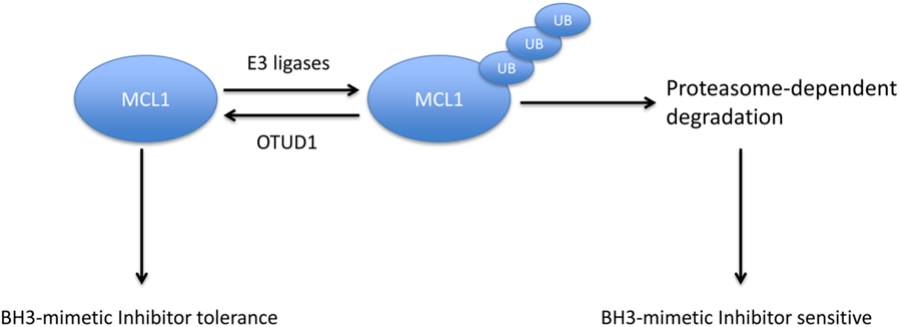
Graphical abstract of our work. MCL1 protein can be ubiquitinated by several E3 ligases and undergo ubiquitination/proteasome-dependent degradation. Our work uncovered that OTUD1 is a deubiquitinating enzyme of MCL1 and regulates the stability of MCL1 protein. Functional studies indicate that OTUD1 antagonizes BH3-mimetic inhibitor induced cell death through targeting MCL1

Using a DUB expression library, we found that OTUD1 regulates the stability of the MCL1 protein. OTUD1 interacts with and promotes the deubiquitination of MCL1 in an enzymatic-activity dependent manner. Through regulating MCL1 stability, OTUD1 regulates the induction of cell death by the BH3-mimetic compound ABT-263 (Fig. 7). According to bioinformatics analysis, we found that OTUD1 is a negative prognostic factor for liver cancer and ovarian cancer and specific subtypes of breast and cervical cancer and that it could be considered as a therapeutic target for curing these cancers.

MCL1 is important for cell proliferation, differentiation and tumourigenesis [27]. MCL1 was initially identified as an immediate early gene expressed during PMA-induced differentiation of myeloid leukaemia cells [28]. Many cells have been shown to rely on MCL1 for their survival and development [29, 30]. The levels of MCL1 are tightly regulated at the transcriptional and post-translational levels [31, 32]. Many cytokines and growth factors, including interleukins and epidermal growth factor, regulate MCL1 transcription in different cells [29]. Moreover, MCL1 is a fast-turnover protein due to ubiquitination by E3 ubiquitin ligases [1–3]. In contrast, two DUBs were uncovered that restrict MCL1 ubiquitination [10, 33]. Here, we found that OTUD1 is a new MCL1 DUB that regulates MCL1 stability and its downstream functions. Our work increases our knowledge about the diverse regulation of MCL1.

There are approximately 95 human DUBs that were classified into five distinct families [5]. Recently, two novel DUB families were uncovered [34, 35]. Among these, there are at least 14 active OTU family deubiquitinases encoded by humans [36]. OTU DUBs play important roles in many signalling pathways including the NF-kB pathway, the interferon pathway, the DNA damage response and so on [37]. OTUD1 belongs to the OTU DUB family and plays important roles in immune responses and breast cancer by regulating different downstream substrates [11, 38–40]. Here, we identified MCL1 as a new substrate of OTUD1. By regulating MCL1 protein levels, OTUD1 regulates cells survival after treatment with the BH3-mimetic compound ABT-263. MCL1 was found to be overexpressed in liver cancer and knockdown of MCL1 sensitizes liver cancer cells to chemotherapy drugs [41]. Moreover, the progression-free survival was poorer among MCL1-positive patients than among MCL1-negative patients with ovarian cancer [25, 42]. Consistently, we found that higher OTUD1 expression was significantly associated with low recurrence-free survival of liver and ovarian cancer patients by analysing the clinical data. Therefore, OTUD1 is a potential target for liver and ovarian cancer therapy. However, the roles of OTUD1 in liver and ovarian cancer are still needed further investigation by using xenograft tumour model, cancer metastasis animal model, and other in vivo cancer models. Moreover, the development of inhibitors of OTUD1 might also help to investigate the role of OTUD1 in vitro and in vivo cancer models.

## Conclusions

Using a DUB expression library, we found that OTUD1 regulates the stability of the MCL1 protein. OTUD1 interacts with and promotes the deubiquitination of MCL1 in an enzymatic-activity dependent manner. Through regulating MCL1 stability, OTUD1 regulates the induction of cell death by the BH3-mimetic compound ABT-263. According to bioinformatics analysis, we found that OTUD1 is a negative prognostic factor for multiple cancers and that it could be considered as a therapeutic target for curing these cancers.

## Supplementary information

**Additional file 1.** Additional figures.

## Data Availability

Please contact corresponding author for data requests.

## Abbreviations

DUB: deubiquitinating enzyme
MCL1: myeloid cell leukaemia 1
OTUD1: OTU domain containing protein 1
BH3: Bcl-2 homology (BH) domain 3
CHX: cycloheximide
