# Supplementary material for: The deubiquitinating enzyme OTUD1 antagonizes BH3-mimetic inhibitor induced cell death through regulating the stability of the MCL1 protein
Source: Cancer Cell Int. 2019 Aug 27;19:222. doi: 10.1186/s12935-019-0936-5 (PMC6712616; doi:10.1186/s12935-019-0936-5)
Supplement: Supplementary file 1 — Additional file 1. Additional figures. [file 12935_2019_936_MOESM1_ESM.ppt]

## Slide 1
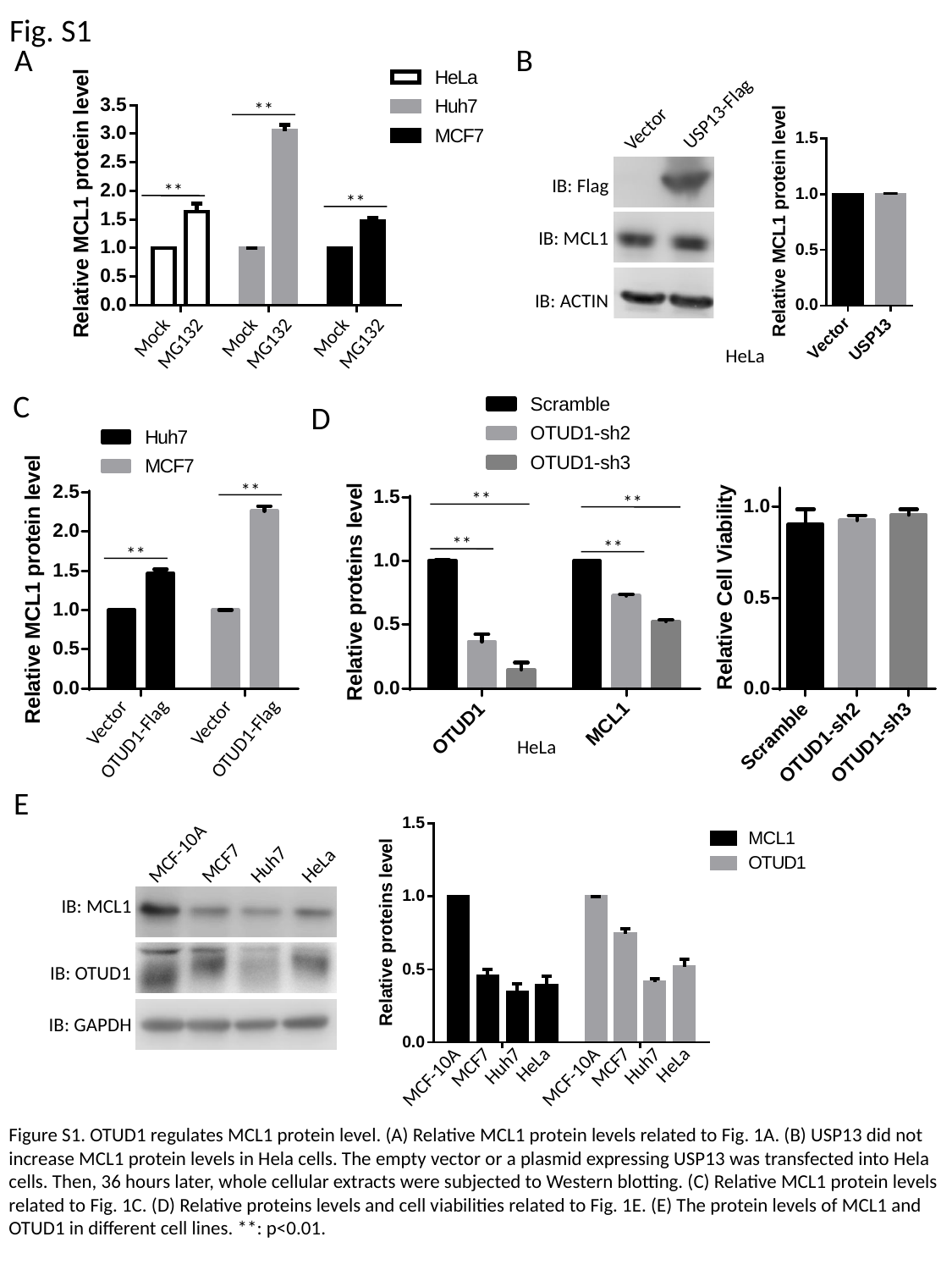

Fig. S1
A
B
USP13-Flag
Vector
IB: Flag
IB: MCL1
IB: ACTIN
**
**
**
Mock
Mock
Mock
MG132
MG132
MG132
HeLa
C
D
**
**
**
**
**
**
Vector
Vector
OTUD1-Flag
OTUD1-Flag
HeLa
E
MCF-10A
MCF7
Huh7
HeLa
IB: MCL1
IB: OTUD1
IB: GAPDH
HeLa
HeLa
Huh7
Huh7
MCF7
MCF7
MCF-10A
MCF-10A
Figure S1. OTUD1 regulates MCL1 protein level. (A) Relative MCL1 protein levels related to Fig. 1A. (B) USP13 did not increase MCL1 protein levels in Hela cells. The empty vector or a plasmid expressing USP13 was transfected into Hela cells. Then, 36 hours later, whole cellular extracts were subjected to Western blotting. (C) Relative MCL1 protein levels related to Fig. 1C. (D) Relative proteins levels and cell viabilities related to Fig. 1E. (E) The protein levels of MCL1 and OTUD1 in different cell lines. **: p<0.01.

## Slide 2
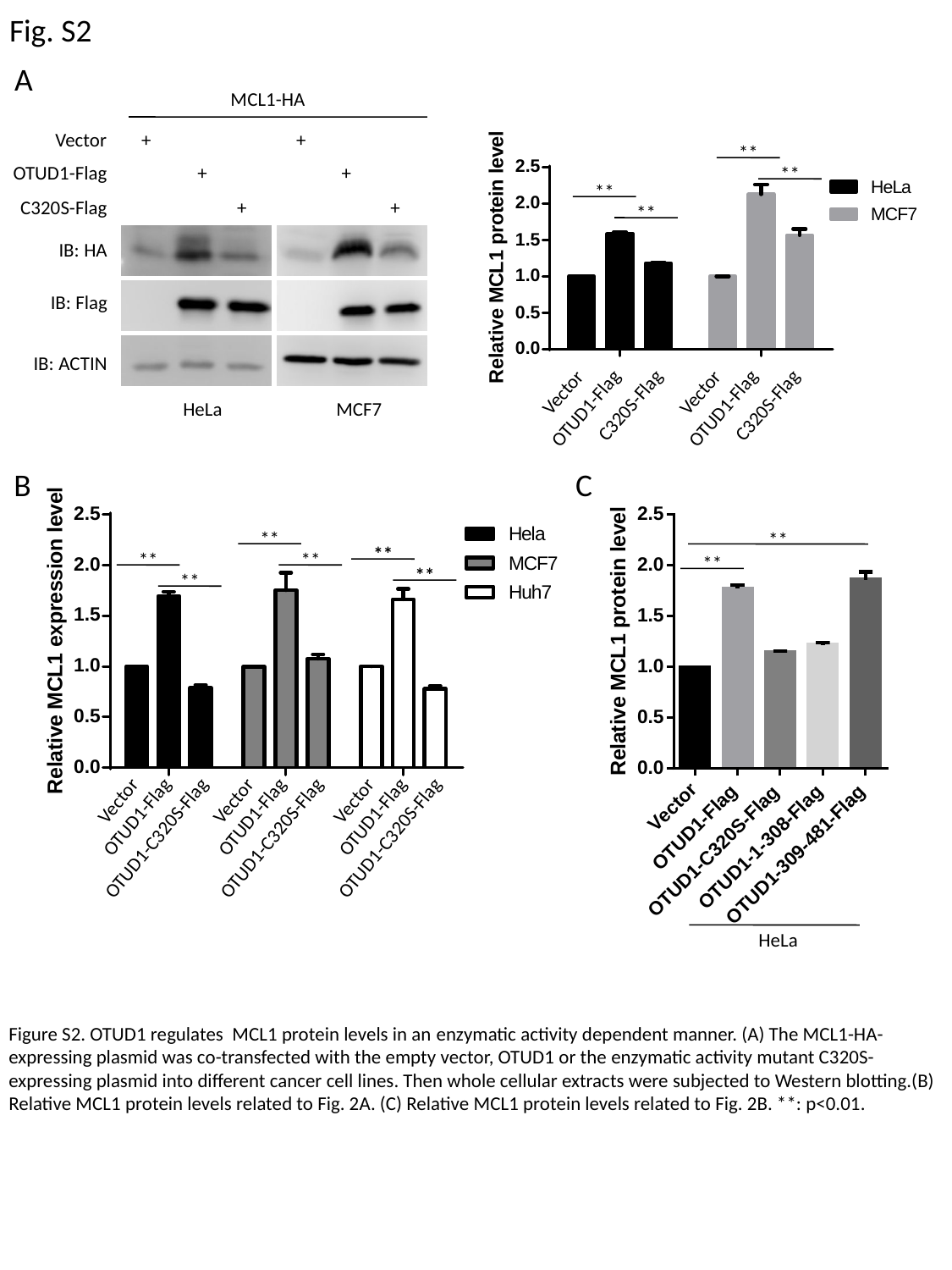

Fig. S2
A
MCL1-HA
Vector
+
+
**
OTUD1-Flag
+
+
**
**
C320S-Flag
+
+
**
IB: HA
IB: Flag
IB: ACTIN
Vector
Vector
C320S-Flag
C320S-Flag
OTUD1-Flag
OTUD1-Flag
HeLa
MCF7
B
C
**
**
**
**
**
**
**
**
Vector
Vector
Vector
OTUD1-Flag
OTUD1-Flag
OTUD1-Flag
OTUD1-C320S-Flag
OTUD1-C320S-Flag
OTUD1-C320S-Flag
HeLa
Figure S2. OTUD1 regulates MCL1 protein levels in an enzymatic activity dependent manner. (A) The MCL1-HA-expressing plasmid was co-transfected with the empty vector, OTUD1 or the enzymatic activity mutant C320S-expressing plasmid into different cancer cell lines. Then whole cellular extracts were subjected to Western blotting.(B) Relative MCL1 protein levels related to Fig. 2A. (C) Relative MCL1 protein levels related to Fig. 2B. **: p<0.01.

## Slide 3
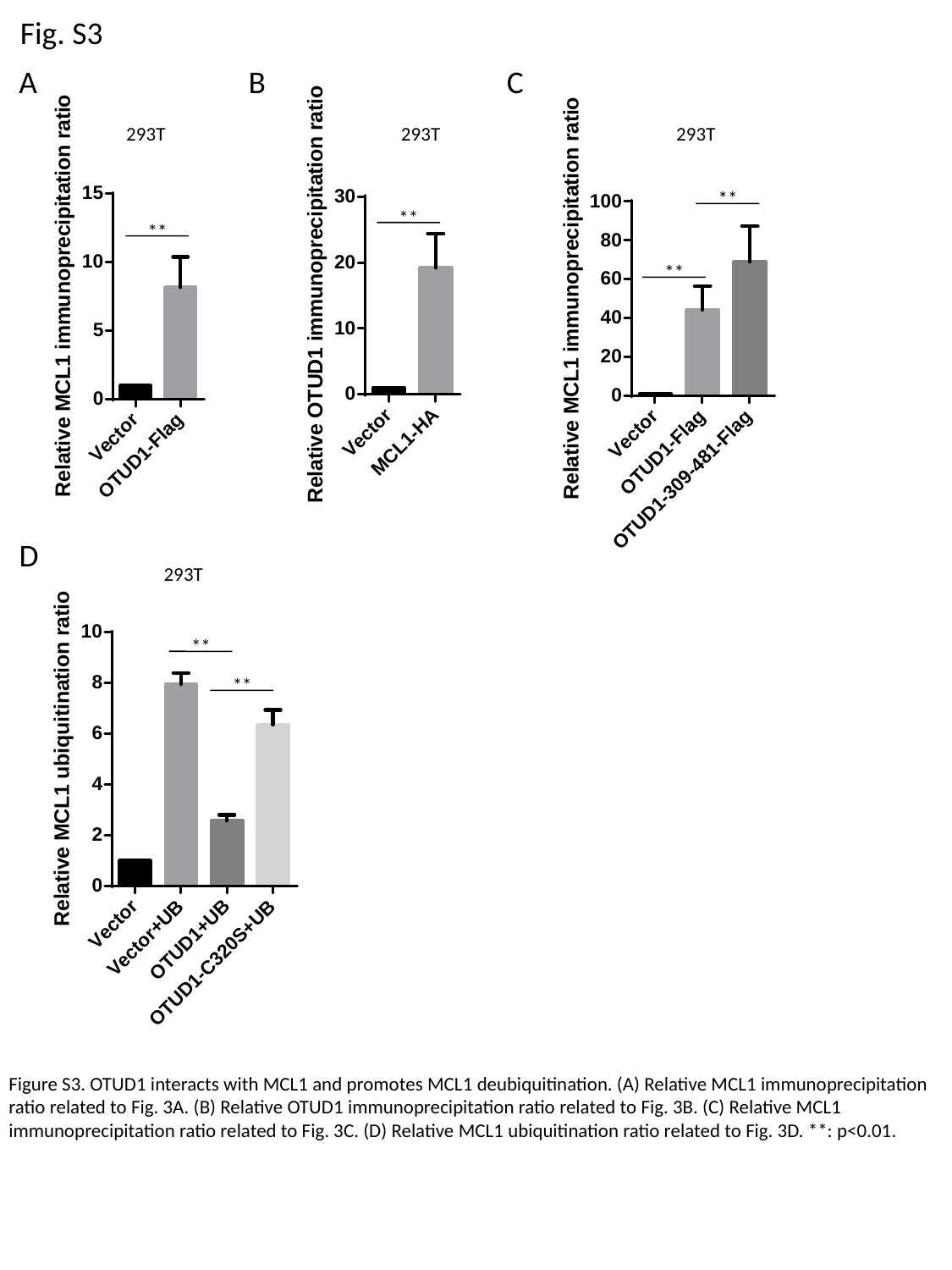

Fig. S3
A
B
C
293T
293T
293T
**
**
**
**
D
293T
**
**
Figure S3. OTUD1 interacts with MCL1 and promotes MCL1 deubiquitination. (A) Relative MCL1 immunoprecipitation ratio related to Fig. 3A. (B) Relative OTUD1 immunoprecipitation ratio related to Fig. 3B. (C) Relative MCL1 immunoprecipitation ratio related to Fig. 3C. (D) Relative MCL1 ubiquitination ratio related to Fig. 3D. **: p<0.01.

## Slide 4
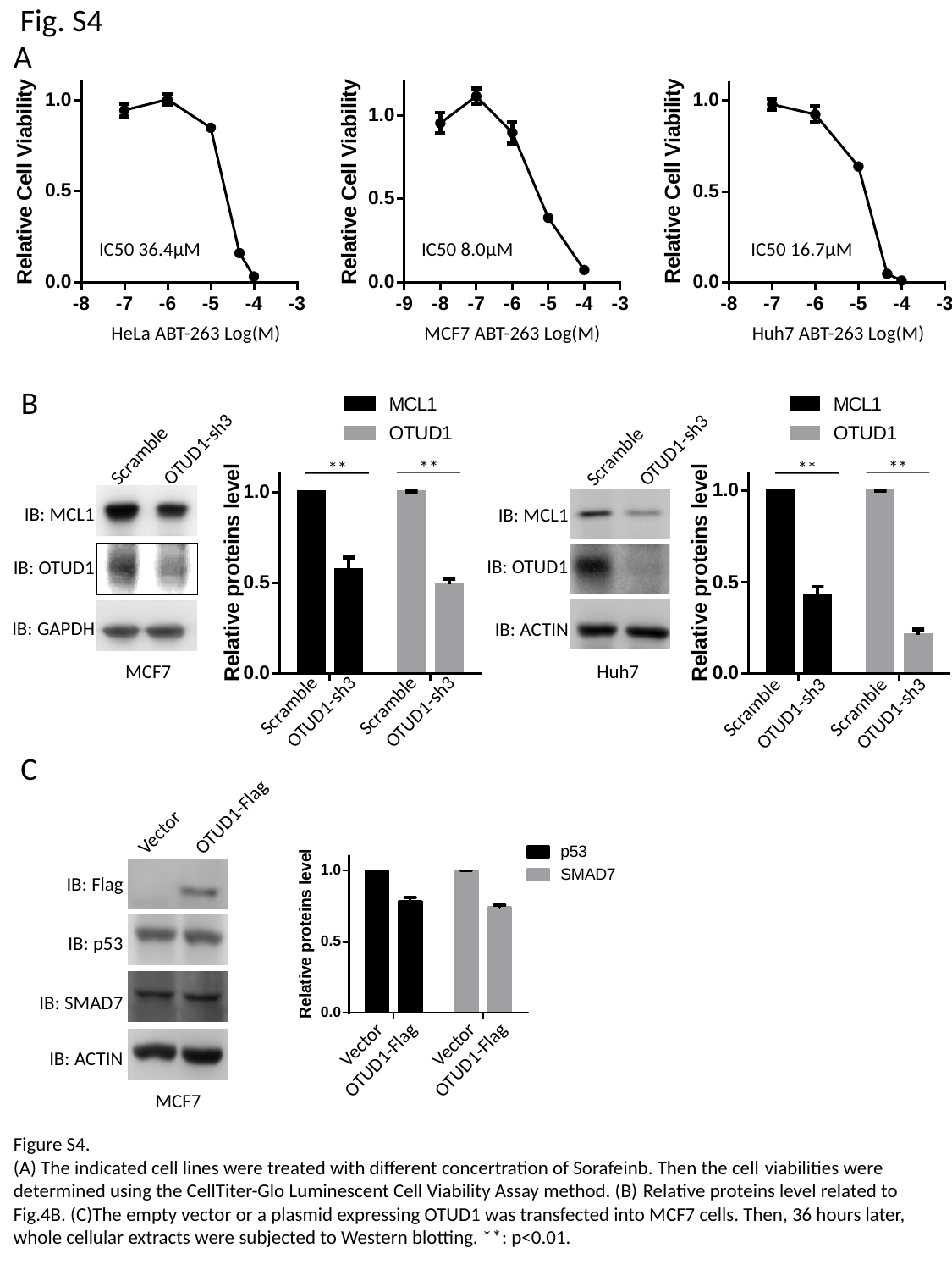

Fig. S4
A
IC50 36.4µM
IC50 8.0µM
IC50 16.7µM
HeLa ABT-263 Log(M)
MCF7 ABT-263 Log(M)
Huh7 ABT-263 Log(M)
B
OTUD1-sh3
OTUD1-sh3
Scramble
Scramble
**
**
**
**
IB: MCL1
IB: MCL1
IB: OTUD1
IB: OTUD1
IB: GAPDH
IB: ACTIN
MCF7
Huh7
Scramble
Scramble
Scramble
Scramble
OTUD1-sh3
OTUD1-sh3
OTUD1-sh3
OTUD1-sh3
C
OTUD1-Flag
Vector
IB: Flag
IB: p53
IB: SMAD7
IB: ACTIN
Vector
Vector
OTUD1-Flag
OTUD1-Flag
MCF7
Figure S4.
(A) The indicated cell lines were treated with different concertration of Sorafeinb. Then the cell viabilities were determined using the CellTiter-Glo Luminescent Cell Viability Assay method. (B) Relative proteins level related to Fig.4B. (C)The empty vector or a plasmid expressing OTUD1 was transfected into MCF7 cells. Then, 36 hours later, whole cellular extracts were subjected to Western blotting. **: p<0.01.

## Slide 5
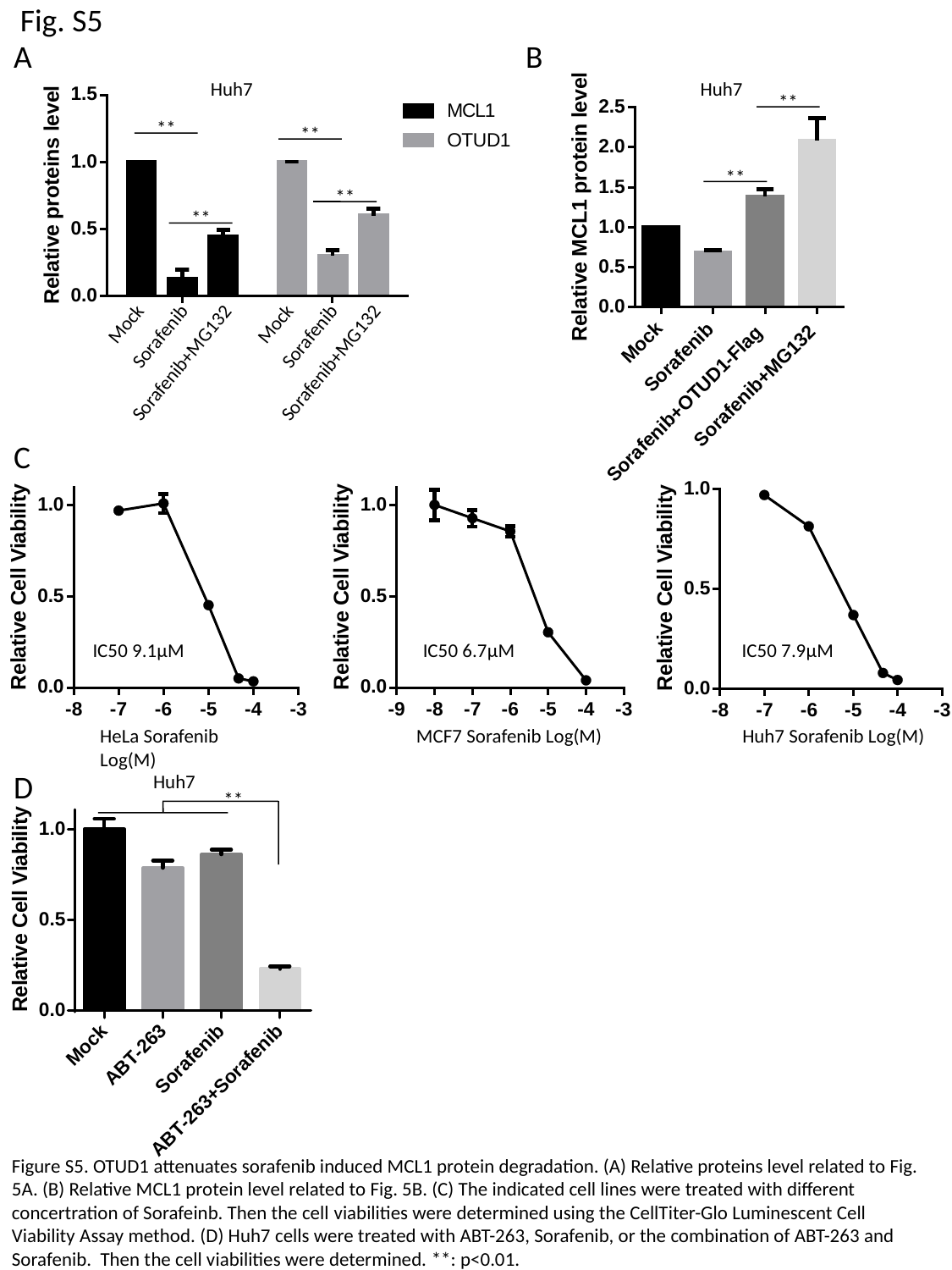

Fig. S5
A
B
Huh7
Huh7
**
**
**
**
**
**
Mock
Mock
Sorafenib
Sorafenib
Sorafenib+MG132
Sorafenib+MG132
C
IC50 9.1µM
IC50 6.7µM
IC50 7.9µM
HeLa Sorafenib Log(M)
MCF7 Sorafenib Log(M)
Huh7 Sorafenib Log(M)
D
Huh7
**
Figure S5. OTUD1 attenuates sorafenib induced MCL1 protein degradation. (A) Relative proteins level related to Fig. 5A. (B) Relative MCL1 protein level related to Fig. 5B. (C) The indicated cell lines were treated with different concertration of Sorafeinb. Then the cell viabilities were determined using the CellTiter-Glo Luminescent Cell Viability Assay method. (D) Huh7 cells were treated with ABT-263, Sorafenib, or the combination of ABT-263 and Sorafenib. Then the cell viabilities were determined. **: p<0.01.
